# Corrigendum: Determining crystal structures through crowdsourcing and coursework

**DOI:** 10.1038/ncomms13392

**Published:** 2016-10-25

**Authors:** Scott Horowitz, Brian Koepnick, Raoul Martin, Agnes Tymieniecki, Amanda A. Winburn, Seth Cooper, Jeff Flatten, David S. Rogawski, Nicole M. Koropatkin, Tsinatkeab T. Hailu, Neha Jain, Philipp Koldewey, Logan S. Ahlstrom, Matthew R. Chapman, Andrew P. Sikkema, Meredith A. Skiba, Finn P. Maloney, Felix R. M. Beinlich, Foldit Players, Ahmet Caglar, Alan Coral, Alice Elizabeth Jensen, Allen Lubow, Amanda Boitano, Amy Elizabeth Lisle, Andrew T. Maxwell, Barb Failer, Bartosz Kaszubowski, Bohdan Hrytsiv, Brancaccio Vincenzo, Breno Renan de Melo Cruz, Brian Joseph McManus, Bruno Kestemont, Carl Vardeman, Casey Comisky, Catherine Neilson, Catherine R. Landers, Christopher Ince, Daniel Jon Buske, Daniel Totonjian, David Marshall Copeland, David Murray, Dawid Jagieła, Dietmar Janz, Douglas C. Wheeler, Elie Cali, Emmanuel Croze, Farah Rezae, Floyd Orville Martin, Gil Beecher, Guido Alexander de Jong, Guy Ykman, Harald Feldmann, Hugo Paul Perez Chan, Istvan Kovanecz, Ivan Vasilchenko, James C. Connellan, Jami Lynne Borman, Jane Norrgard, Jebbie Kanfer, Jeffrey M. Canfield, Jesse David Slone, Jimmy Oh, Joanne Mitchell, John Bishop, John Douglas Kroeger, Jonas Schinkler, Joseph McLaughlin, June M. Brownlee, Justin Bell, Karl Willem Fellbaum, Kathleen Harper, Kirk J. Abbey, Lennart E. Isaksson, Linda Wei, Lisa N. Cummins, Lori Anne Miller, Lyn Bain, Lynn Carpenter, Maarten Desnouck, Manasa G. Sharma, Marcus Belcastro, Martin Szew, Martin Szew, Matthew Britton, Matthias Gaebel, Max Power, Michael Cassidy, Michael Pfützenreuter, Michele Minett, Michiel Wesselingh, Minjune Yi, Neil Haydn Tormey Cameron, Nicholas I. Bolibruch, Noah Benevides, Norah Kathleen Kerr, Nova Barlow, Nykole Krystyne Crevits, Paul Dunn, Paulo Sergio Silveira Belo Nascimento Roque, Peter Riber, Petri Pikkanen, Raafay Shehzad, Randy Viosca, Robert James Fraser, Robert Leduc, Roman Madala, Scott Shnider, Sharon de Boisblanc, Slava Butkovich, Spencer Bliven, Stephen Hettler, Stephen Telehany, Steven A. Schwegmann, Steven Parkes, Susan C. Kleinfelter, Sven Michael Holst, T. J. A. van der Laan, Thomas Bausewein, Vera Simon, Warwick Pulley, William Hull, University of Michigan students, Annes Yukyung Kim, Alexis Lawton, Amanda Ruesch, Anjali Sundar, Anna-Lisa Lawrence, Antara Afrin, Bhargavi Maheshwer, Bilal Turfe, Christian Huebner, Courtney Elizabeth Killeen, Dalia Antebi-Lerrman, Danny Luan, Derek Wolfe, Duc Pham, Elaina Michewicz, Elizabeth Hull, Emily Pardington, Galal Osama Galal, Grace Sun, Grace Chen, Halie E. Anderson, Jane Chang, Jeffrey Thomas Hewlett, Jennifer Sterbenz, Jiho Lim, Joshua Morof, Junho Lee, Juyoung Samuel Inn, Kaitlin Hahm, Kaitlin Roth, Karun Nair, Katherine Markin, Katie Schramm, Kevin Toni Eid, Kristina Gam, Lisha Murphy, Lucy Yuan, Lulia Kana, Lynn Daboul, Mario Karam Shammas, Max Chason, Moaz Sinan, Nicholas Andrew Tooley, Nisha Korakavi, Patrick Comer, Pragya Magur, Quresh Savliwala, Reid Michael Davison, Roshun Rajiv Sankaran, Sam Lee, Saule Tamkus, Shirley Chen, Sho Harvey, Sin Ye Hwang, Sohrab Vatsia, Stefan Withrow, Tahra K Luther, Taylor Manett, Thomas James Johnson, Timothy Ryan Brash, Wyatt Kuhlman, Yeonjung Park, Zoran Popović, David Baker, Firas Khatib, James C. A. Bardwell

Nature Communications 7: Article number:1254910.1038/ncomms12549 (2016); Published 09 16 2016; Updated 10 25 2016

The original version of this Article contained an error in the spelling of a member of the University of Michigan students Consortium, Sam Lee, which was incorrectly given as Sam Lewe. This has now been corrected in both the PDF and HTML versions of the Article.

