## [Peer Review File · Nature Communications]

Reviewer #1 (Remarks to the Author):

The manuscript by Horowitz et al. describes a new feature of the citizen science game Foldit using electron density maps. In this version, the goal of the users is to fit the protein structure to a density map. The authors organized a competition between 2 crystallographers, 469 Foldit players, 58 undergraduate students playing the game as part of a biology class at U. of Michigan, and 2 programs (Phenix Autosolve and MR-Rosetta). At the end of this competition, a team of Foldit players reported the most accurate structure. In order to obtain this structure, the authors pruned the models generated by Foldit players to include only residues modeled by the crystallographers. The analysis of this structure contributed to the discovery of new family of histidine triad proteins, which has been tested in-vitro to reduce amyloid aggregation.

The paper is very well written and documented. The results and observations reported in this manuscript are interesting and provide useful insights into the performance of crowd-computing systems. The discovery of a novel protein family is also a strong asset of this manuscript.

Major Comments:

- The paper emphasizes the participation of undergraduate students to the competition. I found this experiment interesting and instructive. However, it seems that this group had the lowest performance (?) Therefore, it raises the question of the necessity to include this group in the competition. To motivate their presence, it could have been interesting to include data (from a survey?) about their perception of difficulties, understanding of core concepts, etc.
- L. 65-66: "we pruned the Foldit models afterwards to include only those residues modeled by the top trained crystallographer" I agree that Foldit players were initially disadvantaged, but if the residues included/removed have been selected based on the knowledge/expertise of the crystallographers, it seems to me difficult to claim that Foldit players alone performed better than crystallographers. It may be simply my misunderstanding of the protocol used here, but in any cases it would be nice to clarify this point/sentence.
- L. 67: Similarly, "We then re-refined the pruned Foldit structures" How did the authors refine these structures (I probably have missed it)? If computer programs are required to obtain the improved structure, it should be emphasized in the manuscript. It does not alter at all the value of the results, but it would be helpful to clarify it here again.
- A fixed amount of time was allocated to all participants (crystallographers, Foldit players, students and computer programs). However, this time does not reflect the time effectively spent at solving the problem. If possible, it would be interesting to include these numbers (i.e. time spent working on the puzzle) to give us a clearer perspective of the efficiency of each group.
- The competition was conducted with a single structure. I do acknowledge the difficulty to conduct another similar experiment for this paper (especially with the same impact). However, it would be helpful to understand if the results are reproducible with other structures, or what is the "average" magnitude of the improvement. I guess that the authors may have already accumulated data for other molecules, and if that were the case it would be helpful to add this information in the supplementary material.

Minor comments:

- L. 44: "Many of the Foldit players, in contrast, had attempted to solve early electron density puzzles" Can the authors report how many of them effectively used the density map before?
- L. 104-105: "We envision a future in which professional crystallographers frequently tap the collective model building expertise of Foldit players." Are some systems already in place? What is the availability of this technique to the community?
- YPL067C is the first member of a new protein family. I was interested to know if the choice of YPL067C has been dictated by a prior knowledge suggesting that it could be the case (and in that case some structural features characterizing this family could have been known before the experiment), or if the new Foldit structure revealed unexpected findings.
- L. 139-140: "To facilitate work on electron density data in Foldit, new visualizations and tools, along with a tutorial puzzle to introduce them" Same as above. Can the authors report how many

times this tutorial has been used (was it mandatory?) ?

Reviewer #2 (Remarks to the Author):

In this manuscript, the authors described a feat achieved by crowdsourcing using Foldit on refinement of protein crystallography model-building. In the competition on protein YPL067C, the (relatively) amateur Foldit players' top model rivals and even surpasses the answer by experts in crystallography in all measures, after pruning out low-density regions. Considering that the target protein lacks close homologs in PDB, the results shed light on the potential of crowdsourcing in crystal structure refinement.

This work has high significance and is suitable for publication. Crowdsourcing in biological research is a powerful trend, as stated by the authors. I would recommend for minor revision. With the following comments addressed, the manuscript would be even more remarkable:

The exclusion of poor-density regions turned defeat into victory for Foldit models. The author mentioned that the current version of Foldit allows no gaps thus the players are at disadvantage. An addition of such feature would be great for Foldit and its further application. Furthermore, the decision-making on which regions to exclude is also an important part of model refinement. Thus, it would serve as a better test for the power of crowdsourcing.

In other crowdsourcing projects, e.g. eterna for RNA folding, the insights provided by players are invaluable and inspired hypotheses that have not been considered before. So what are the Foldit players "secrets" to beat the crystallographers? A "postmortem" reflection may reveal some of the key decisions made by players that lead to success. Also, it would be interesting to know if such players' "tips" are simply unheard of by experts. Is it possible to incorporate the players' insight to make better automated refinement programs?

On the other hand, the manuscript mentioned the attribution of better side-chain conformations, but with no details shown. Some zoom-in views on differences of side-chain examples would help visualize the subtlety.

Last, the author could describe the overall difficulty level for refinement of YPL067C. And what does the distribution of all 1094 Foldit models look like? Additionally, it would be interesting to show the distribution of players' ranks/scores against the quality of their submissions. It is expected that the top players contribute the most, and such "growth curve" would reveal the power of the crowd.

Minor Comments

1. On line 304, appearance of "COOT" in all-cap seems inconsistent with the rest of manuscript.
2. The supplementary text mentioned more results on lectin scytovirin on Foldit players and students. Summary of statistics and example structure models would be helpful to evaluate those.

Reviewer #3 (Remarks to the Author):

This is an interesting paper, in principle suitable for publication in Nature Communications. I have one serious problem with the method described. As the authors show, the widely used program

Phenix Autosolve produces an excellent solution of this structure entirely automatically, and this is probably currently the method of choice for solving such a

structure by selenomethionine phasing. As explained starting at line 157, Phenix Autosolve with model building switched off was used to obtain 'experimental' electron density. If I have understood correctly, the map was then set to zero at all points more than 5 Angstroms from the Phenix Autosolve solution (obtained in a separate run including model building). In my opinion this must enormously simplify the early stages of tracing the map by hand (or by using foldit). I would have been much more impressed if the structure could be solved directly by crowd sourcing without this substantial assistance from Phenix Autosolv. A better test would have been to give the students etc. the UNMASKED experimental density from Phenix Autosolve to trace; I presume that this was tried but failed.

Despite this, it is interesting that, judging by Figure 2, the crowd sourcing solution is not so much worse than the fully automatic Phenix Autosolve solution. I think that this tells us that Phenix Autosolve still has something to learn! I suspect that the standard approach by of first tracing the main chain and then fitting the side-chains could be improved by giving more weight to the positioning of the aromatic residues, as the students probably did intuitively. This reminds me of the recent unexpected success by a program beating the GO world champion: programs can learn too, So I suspect that crowd sourcing is an interesting academic exercise but has little future as a practicable way of solving structures, because the automated programs will win in the long run, possibly by learning new tricks from crowd sourcing!

The paper is also interesting from a structural biology point of view.

Reviewer 1

The manuscript by Horowitz et al. describes a new feature of the citizen science game Foldit using electron density maps. In this version, the goal of the users is to fit the protein structure to a density map. The authors organized a competition between 2 crystallographers, 469 Foldit players, 58 undergraduate students playing the game as part of a biology class at U. of Michigan, and 2 programs (Phenix Autosolve and MR-Rosetta). At the end of this competition, a team of Foldit players reported the most accurate structure. In order to obtain this structure, the authors pruned the models generated by Foldit players to include only residues modeled by the crystallographers. The analysis of this structure contributed to the discovery of new family of histidine triad proteins, which has been tested in-vitro to reduce amyloid aggregation.

The paper is very well written and documented. The results and observations reported in this manuscript are interesting and provide useful insights into the performance of crowd-computing systems. The discovery of a novel protein family is also a strong asset of this manuscript.

We thank the reviewer for his/her kind comments.

Major Comments:

- The paper emphasizes the participation of undergraduate students to the competition. I found this experiment interesting and instructive. However, it seems that this group had the lowest performance (?) Therefore, it raises the question of the necessity to include this group

in the competition. To motivate their presence, it could have been interesting to include data (from a survey?) about their perception of difficulties, understanding of core concepts, etc. *The students worked hard to win the competition, so we feel honor-bound to report their standings, as is the tradition in sporting events, for instance. We also think that they were an interesting and important part of the competition, as our previous observations that undergraduate students could improve a published crystal structure is what directly led to this study. As a result, the most obvious question was whether the students could do similarly well with the more difficult model-building problem. To make the necessity of the students to be part of the competition more clear, we have moved the supplementary introduction section describing this previous assignment to the main text:*

Recently, in a class assignment, we asked 57 undergraduate students to build the structure of a protein, lectin scytovirin³, using only the model-building program Coot and an electron density map downloaded from the electron density server⁴. Students were not given the amino acid sequence of the protein, but were provided with the position of the N-terminal amino acid. The students were instructed to build the structure of the protein, residue-by-residue, into the $2F_o-F_c$ electron density map. Many students expressed appreciation for the puzzle-like quality of the assignment. In addition to learning about protein structure, ~10% of these students improved upon the previously published model⁴. One student even generated a structure that ranked in the 100th percentile in both Molprobity clashscore and total score when compared to other structures in its resolution range. These results raised the intriguing possibility that even a relatively small group of amateur model builders could collectively build higher quality models than a single trained crystallographer. This concept was remarkably reminiscent of ideas recently championed by the online protein-folding computer game Foldit.

and have increased the discussion of how the two assignments differed:

In our previous study, students built into a high-resolution, fully refined map³. In the study presented here, students received a lower resolution, unrefined map, and no starting place for building.

- L. 65-66: "we pruned the Foldit models afterwards to include only those residues modeled by the top trained crystallographer" I agree that Foldit players were initially disadvantaged, but if the residues included/removed have been selected based on the knowledge/expertise of the crystallographers, it seems to me difficult to claim that Foldit players alone performed better than crystallographers. It may be simply my misunderstanding of the protocol used here, but in any cases it would be nice to clarify this point/sentence.

We did not mean to suggest that the victory came from Foldit players alone. We do not think that removal of crystallographers from the process of crystallography is a good idea. Instead, we see many possibilities for productive collaborative interactions between crystallographers and Foldit players. A top priority for future Foldit development is making it possible for the

Foldit players to remove residues, and to have the Foldit score accurately reward the removal of disordered residues. We have added a discussion of this point to the discussion:

To further improve the capability for Foldit players to aid crystallographers, ongoing development will make it possible for the players to add or remove residues with insufficient electron density, and have these changes accurately reflected in the Foldit score.

- L. 67: Similarly, "We then re-refined the pruned Foldit structures" How did the authors refine these structures (I probably have missed it)? If computer programs are required to obtain the improved structure, it should be emphasized in the manuscript. It does not alter at all the value of the results, but it would be helpful to clarify it here again.

The procedure of doing automated Phenix refinement on all the competition structures to correct trivial errors was previously mentioned, but only in the methods, but we have now also mentioned it in the results section to help increase clarity of this point:

To determine which group produced the best structural models, we used an automated refinement procedure¹² on all the structures, and then compared key crystallographic statistics.

- A fixed amount of time was allocated to all participants (crystallographers, Foldit players, students and computer programs). However, this time does not reflect the time effectively spent at solving the problem. If possible, it would be interesting to include these numbers (i.e. time spent working on the puzzle) to give us a clearer perspective of the efficiency of each group.

The trained crystallographers spent between 8 and 14 hours on the assignment. Several students noted upon turning in the assignment that they spent around 30-40 hours on the assignment, however, we do not know statistics on exactly how long they spent. Similarly, we are not able to recover exactly how long the Foldit players spent on the puzzle, but were able to determine that the winning team did start the puzzle near the beginning of the competition, and finished it a few days before the end. These details have been added in the methods:

The puzzle was open to Foldit players for 28 days. Members of the winning team began playing the day after the puzzle opened, and produced the winning structure approximately 23 days later—four days before the puzzle closed.

The trained crystallographers spent approximately eight and fourteen hours, respectively, working on the puzzle.

- The competition was conducted with a single structure. I do acknowledge the difficulty to conduct another similar experiment for this paper (especially with the same impact). However, it would be helpful to understand if the results are reproducible with other structures, or what is the "average" magnitude of the improvement. I guess that the authors

may have already accumulated data for other molecules, and if that were the case it would be helpful to add this information in the supplementary material.

We have not accumulated more data of this type currently stored away. Identifying, crystallizing, and solving the crystal structure of one of those increasingly rare proteins that contains no recognizable sequence identity to those in the PDB, and then running a subsequent Foldit puzzle is beyond the scope of this paper.

Minor comments:

- L. 44: "Many of the Foldit players, in contrast, had attempted to solve early electron density puzzles" Can the authors report how many of them effectively used the density map before?

Based on the histories of the players who tried the puzzle, 54% of players had previously played electron density puzzles. This is now stated in the main text:

In contrast, 54% of the participating Foldit players had attempted to solve early electron density puzzles in Foldit and thus had some experience in Foldit-based model building.

- L. 104-105: "We envision a future in which professional crystallographers frequently tap the collective model building expertise of Foldit players." Are some systems already in place? What is the availability of this technique to the community?

The Foldit team currently welcomes any and all crystallographic collaborators. Prior to this proof-of-principle study, it was not widely advertised that crystallographers should use Foldit, as we first wanted to carefully examine its utility. We anticipate that the publication of this success story in a high-impact journal will definitely raise the awareness and interest in the crystallographic community to utilize Foldit. Upon publication of this paper, we will highlight it on the Foldit website and will advertise the continued search for collaborations on crystallography mailing lists.

- YPL067C is the first member of a new protein family. I was interested to know if the choice of YPL067C has been dictated by a prior knowledge suggesting that it could be the case (and in that case some structural features characterizing this family could have been known before the experiment), or if the new Foldit structure revealed unexpected findings.

In short, no. Before running the competition, we were aware of the published phenotype of this protein, but had no other insight into its structure or function from homology searches. Based off of the lack of information available in the homology searches, we thought it likely that YPL067C represented a new protein family. However, we had no idea what the structure and distinguishing features would be prior to solving the structure.

- L. 139-140: "To facilitate work on electron density data in Foldit, new visualizations and tools, along with a tutorial puzzle to introduce them" Same as above. Can the authors report how many times this tutorial has been used (was it mandatory?) ?

While not technically mandatory, it is highly likely that all the players did use the tutorial, as

the game persistently reminds you to do so whenever an electron density puzzle is opened if you have not played the tutorial. However, as the game does not track the use of the tutorial, we are not able to report on the extent it was used.

Reviewer #2 (Remarks to the Author):

In this manuscript, the authors described a feat achieved by crowdsourcing using Foldit on refinement of protein crystallography model-building. In the competition on protein YPL067C, the (relatively) amateur Foldit players' top model rivals and even surpasses the answer by experts in crystallography in all measures, after pruning out low-density regions. Considering that the target protein lacks close homologs in PDB, the results shed light on the potential of crowdsourcing in crystal structure refinement.

This work has high significance and is suitable for publication. Crowdsourcing in biological research is a powerful trend, as stated by the authors. I would recommend for minor revision. With the following comments addressed, the manuscript would be even more remarkable:

We thank the reviewer for his/her positive feedback.

The exclusion of poor-density regions turned defeat into victory for Foldit models. The author mentioned that the current version of Foldit allows no gaps thus the players are at disadvantage. An addition of such feature would be great for Foldit and its further application. Furthermore, the decision-making on which regions to exclude is also an important part of model refinement. Thus, it would serve as a better test for the power of crowdsourcing.

As discussed in response to Reviewer 1, we definitely agree that the ability for players to remove residues of disorder and have it accurately reflected in the Foldit score is a top priority for future puzzles. We are beginning to implement our first attempts, and now discuss this goal near the end of the paper:

To further improve the capability for Foldit players to aid crystallographers, ongoing development will make it possible for the players to add or remove residues with insufficient electron density, and have these changes accurately reflected in the Foldit score.

In other crowdsourcing projects, e.g. eterna for RNA folding, the insights provided by players are invaluable and inspired hypotheses that have not been considered before. So what are the Foldit players "secrets" to beat the crystallographers? A "postmortem" reflection may reveal some of the key decisions made by players that lead to success. Also, it would be interesting to know if such players' "tips" are simply unheard of by experts. Is it possible to incorporate the players' insight to make better automated refinement programs?

The results of the competition undoubtedly should influence the overall strategy employed by crystallographers. We have added a new figure (Supplementary Figure 3), in which we break down the types of moves used by the players, which should be helpful to guide both automated and manual building and refinement strategies. Furthermore, we have included a

new suggestion in the discussion that collaborative work on model building and refinement may be beneficial:

The collaborative building process used by the Foldit players could also be a beneficial strategy for professional crystallographers, who could achieve a similar effect by either having multiple laboratory members take turns working on model building and refinement, or by submitting their crystal refinement problems to Foldit. Looking forward, we hope that further analysis of electron density puzzle solutions in Foldit can inform continued improvement of automated structure solution algorithms.

On the other hand, the manuscript mentioned the attribution of better side-chain conformations, but with no details shown. Some zoom-in views on differences of side-chain examples would help visualize the subtlety.

We have added a Supplementary Figure 5 to provide some examples of differing side chain conformations between the best crystallographer and Foldit structures.

Last, the author could describe the overall difficulty level for refinement of YPL067C.

The following sentences have been added to the methods:

When asked to describe the difficulty level of this assignment, the trained crystallographers rated it as somewhat difficult (on a scale of: very difficult, somewhat difficult, neither easy nor difficult, somewhat easy, very easy).

And what does the distribution of all 1094 Foldit models look like? Additionally, it would be interesting to show the distribution of players' ranks/scores against the quality of their submissions. It is expected that the top players contribute the most, and such "growth curve" would reveal the power of the crowd.

We have added Supplementary Figure 6 (pasted below), which shows the distribution of Foldit scores in the competition to address this question. The graph clearly shows that the best scoring puzzles resulted in the highest quality structures after refinement.

Supplementary Figure 6 | Foldit model score distribution and relation to R_{free} . (a) After clustering the >900,000 Foldit models by structural alignment (with a cluster radius of 1 Å RMSD), Phenix refinement was carried out with the 1000 best-scoring cluster centers, along with the 50 best-scoring solo or team models and any models that were flagged by Foldit players for special consideration—1094 Foldit models in total. The most favorable Foldit scores (lowest Rosetta energy) were associated with low R_{free} values. (b) The score distribution of Foldit models is strongly bimodal, suggesting that Rosetta scoring can effectively discriminate models that correctly fit the electron density map. Experienced Foldit players performed best in this puzzle. Among all 469 participants of the Foldit puzzle, each player had previously played an average of 10 Foldit puzzles with electron density; among the seven soloist players that achieved a Rosetta energy less than -700, each player averaged 39 previous electron density puzzles.

Minor Comments

1. On line 304, appearance of "COOT" in all-cap seems inconsistent with the rest of manuscript.

This mistake has been changed in the text:

Iterative refinement and model building was performed using Phenix Refine¹² and Coot¹¹.

2. The supplementary text mentioned more results on lectin scytovirin on Foldit players and students. Summary of statistics and example structure models would be helpful to evaluate those.

We have included more details on the results of the lectin scytovirin puzzle in this version to help clarify some of our choices in the subsequent competition. Most notably, we have included a new figure (Supplementary Figure 2), which shows how the lectin scytovirin puzzle guided our choice of electron density map trimming.

Reviewer #3 (Remarks to the Author):

This is an interesting paper, in principle suitable for publication in Nature Communications.

We thank the reviewer for their positive outlook.

I have one serious problem with the method described. As the authors show, the widely used program Phenix Autosolve produces an excellent solution of this structure entirely automatically, and this is probably currently the method of choice for solving such a structure by selenomethionine phasing. As explained starting at line 157, Phenix Autosolve with model building switched off was used to obtain 'experimental' electron density. If I have understood correctly, the map was then set to zero at all points more than 5 Angstroms from the Phenix Autosolve solution (obtained in a separate run including model building). In my opinion this must enormously simplify the early stages of tracing the map by hand (or by using foldit). I would have been much more impressed if the structure could be solved directly by crowd sourcing without this substantial assistance from Phenix Autosolve. A better test would have been to give the students etc. the UNMASKED experimental density from Phenix Autosolve to trace; I presume that this was tried but failed.

The reviewer raises a very interesting point that was not specifically addressed in our original manuscript: how much did our trimming the map affect the ability for the students and Foldit players to build the structure accurately? Thankfully, we tested this facet in the preliminary lectin scytovirin puzzle, in which the students and Foldit players were both given the entire unit cell to build into, and not just a single monomer. In this previous iteration, almost all the students built only into the density of a single monomer without having the model cross into density from other monomers, and the top nine Foldit solutions also only built into the electron density of single monomers. We have now added Supplementary Figure 2, which discusses this observation. Given this success rate in the previous iteration, we considered that trimming the map to just represent one monomer would probably not substantially affect the results of this competition. As we wanted to encourage many Foldit players to try this puzzle, and this protein and map were a little larger than the usual Foldit puzzles, we had trimmed the map to help increase playability for Foldit players worldwide including those with slightly older computers.

Despite this, it is interesting that, judging by Figure 2, the crowd sourcing solution is not so much worse than the fully automatic Phenix Autosolve solution. I think that this tells us that Phenix Autosolve still has something to learn! I suspect that the standard approach by of first tracing the main chain and then fitting the side-chains could be improved by giving more weight to the positioning of the aromatic residues, as the students probably did intuitively. This reminds me of the recent unexpected success by a program beating the GO world champion: programs can learn too, So I suspect that crowd sourcing is an interesting academic exercise but has little future as a practicable way of solving structures, because the automated programs will win in the long run, possibly by learning new tricks from crowd sourcing!

Reviewer 2 also made a very similar point, as follows:

“In other crowdsourcing projects, e.g. eterna for RNA folding, the insights provided by players are invaluable and inspired hypotheses that have not been considered before. So what are the Foldit players "secrets" to beat the crystallographers? A "postmortem" reflection may reveal some of the key decisions made by players that lead to success. Also, it would be interesting to know if such players' "tips" are simply unheard of by experts. Is it possible to incorporate the players' insight to make better automated refinement programs?”

We agree with both reviewers that in the long run, automated, crowdsourced, and hand-building techniques by experts will all learn and grow together. We are hopeful that the results of the competition will influence the overall strategy employed by crystallographers and crystallographic programs. We have added a new figure (Supplementary Figure 3), in which we break down the types of moves used by the players, which should be helpful to guide both automated and manual building and refinement strategies. Similarly, we have added a note on the potential benefits of collaborative model building and refinement:

The collaborative building process used by the Foldit players could also be a beneficial strategy for professional crystallographers, who could achieve a similar effect by either having multiple laboratory members take turns working on model building and refinement, or by submitting their crystal refinement problems to Foldit. Looking forward, we hope that further analysis of electron density puzzle solutions in Foldit can inform continued improvement of automated structure solution algorithms.

Many of the Foldit players were more than happy to share their tips and strategies with us, which guided our description of the method they used to build the structure. As new puzzles with new challenges are introduced, we anticipate that the Foldit players will be more than happy to share their methodological advances with the wider scientific community.

The paper is also interesting from a structural biology point of view.

We thank the reviewer for this positive feedback. We had tried very hard to figure out what family of proteins YPL067C belonged to prior to the solution of the structure, and we had come up relatively empty, with only fungal proteins of unknown function and structure. The solution of the structure revealed YPL067C to be the founding member of the HTC family within the broader superfamily of HIT proteins. Prior to this work, there was no indication that any HIT proteins might be involved in prevention of amyloid toxicity. In the end, we believe this information makes this manuscript especially valuable, as it substantially broadens the biological impact of the findings.

Reviewer #1 (Remarks to the Author):

The authors answered all my comments. I believe that this work will be of interest for the broad readership of Nature Communication. In particular, I do agree with the authors that "... the publication of this success story in a high-impact journal will definitely raise the awareness and interest in the crystallographic community to utilize Foldit."

Reviewer #2 (Remarks to the Author):

The authors have addressed my remarks satisfactorily. They have done a good job adding more information as well as text to clarify important procedures. The summary of player moves and score distribution is helpful for deeper analysis. The manuscript is suitable for publication in my point of view.

REVIEWERS' COMMENTS:

Reviewer #1 (Remarks to the Author):

The authors answered all my comments. I believe that this work will be of interest for the broad readership of Nature Communication. In particular, I do agree with the authors that "... the publication of this success story in a high-impact journal will definitely raise the awareness and interest in the crystallographic community to utilize Foldit."

We thank the reviewer for for his/her kind comments.

Reviewer #2 (Remarks to the Author):

The authors have addressed my remarks satisfactorily. They have done a good job adding more information as well as text to clarify important procedures. The summary of player moves and score distribution is helpful for deeper analysis. The manuscript is suitable for publication in my point of view.

We are glad that the reviewer approves of the new changes.