# Supplementary material for: Determining crystal structures through crowdsourcing and coursework
Source: Nat Commun. 2016 Sep 16;7:12549. doi: 10.1038/ncomms12549 (PMC5028414; doi:10.1038/ncomms12549)
Supplement: Supplementary Information — Supplementary Figures 1-10, Supplementary Table 1, Supplementary Notes 1-2 and Supplementary References. [file ncomms12549-s1.pdf]

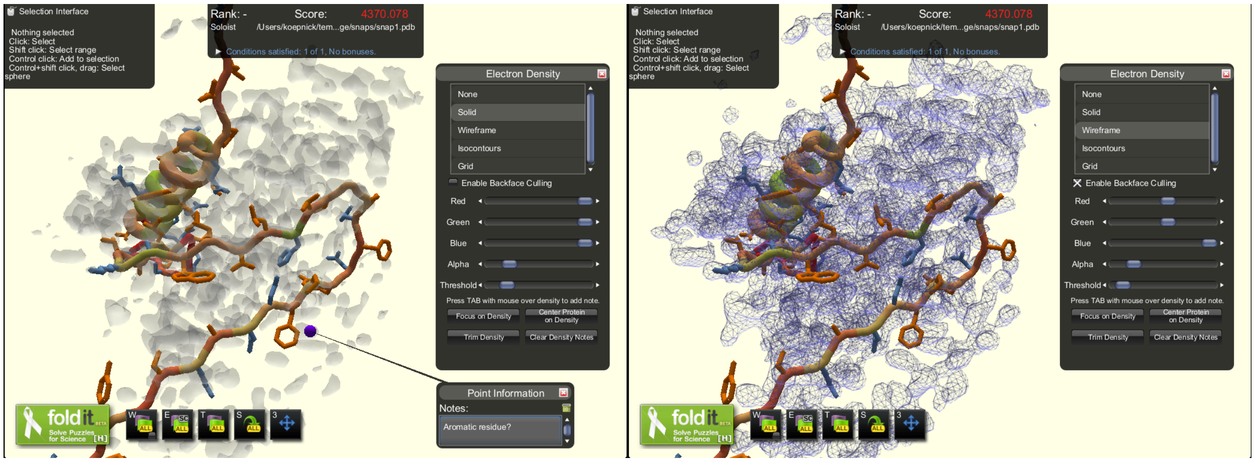

Supplementary Figure 1 | Electron density visualization in Foldit. Electron density rendered as solid (left) or wireframe (right), which can be selected from four different visualization options in the Electron Density menu (right portion of each panel). The Electron Density menu also contains controls for viewing adjustment, including changing color, trimming density, and changing map threshold. Adding notes to specific points in the density (left panel, bottom) can aid in model building and refinement.

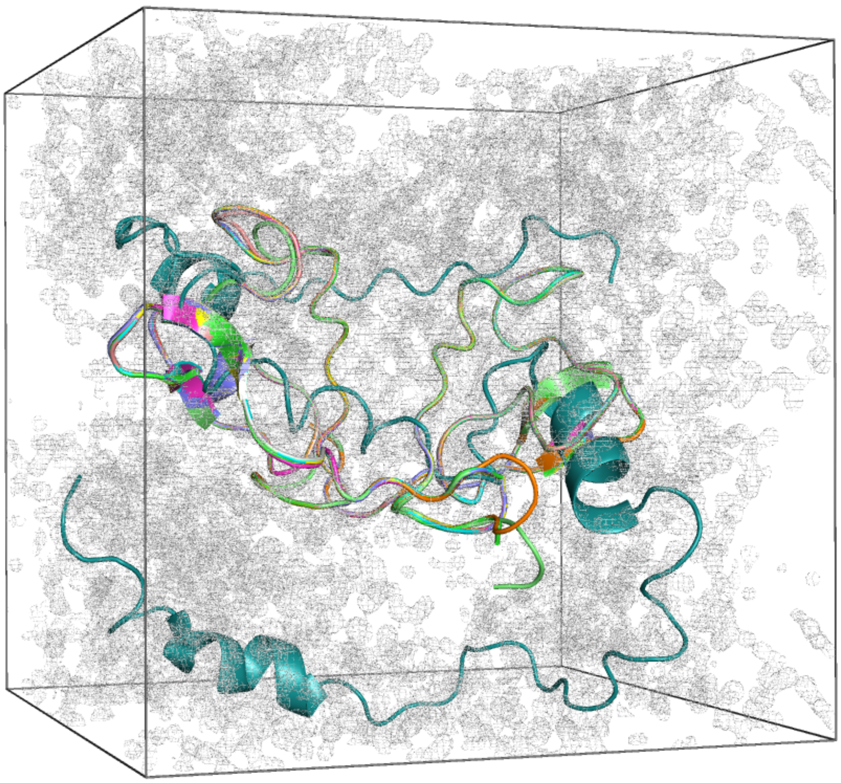

Supplementary Figure 2 | Lectin scytovirin top ten Foldit models overlaid in unit cell density. In a previous challenge, Foldit players were provided with the complete $P2_12_12_1$ unit cell of lectin scytovirin crystal density (PDB ID: 2QT4). The top nine Foldit models (ranked by Foldit score) were all correctly placed within the density of a single monomer in the unit cell. The tenth-ranked structure (deep teal) bridged density from several symmetric copies of protein in the unit cell. Based on the success of the Foldit players in the lectin scytovirin puzzle in using only the electron density from one monomer, the density map for YPL067C was masked to include only a portion of the unit cell comprising a single monomer.

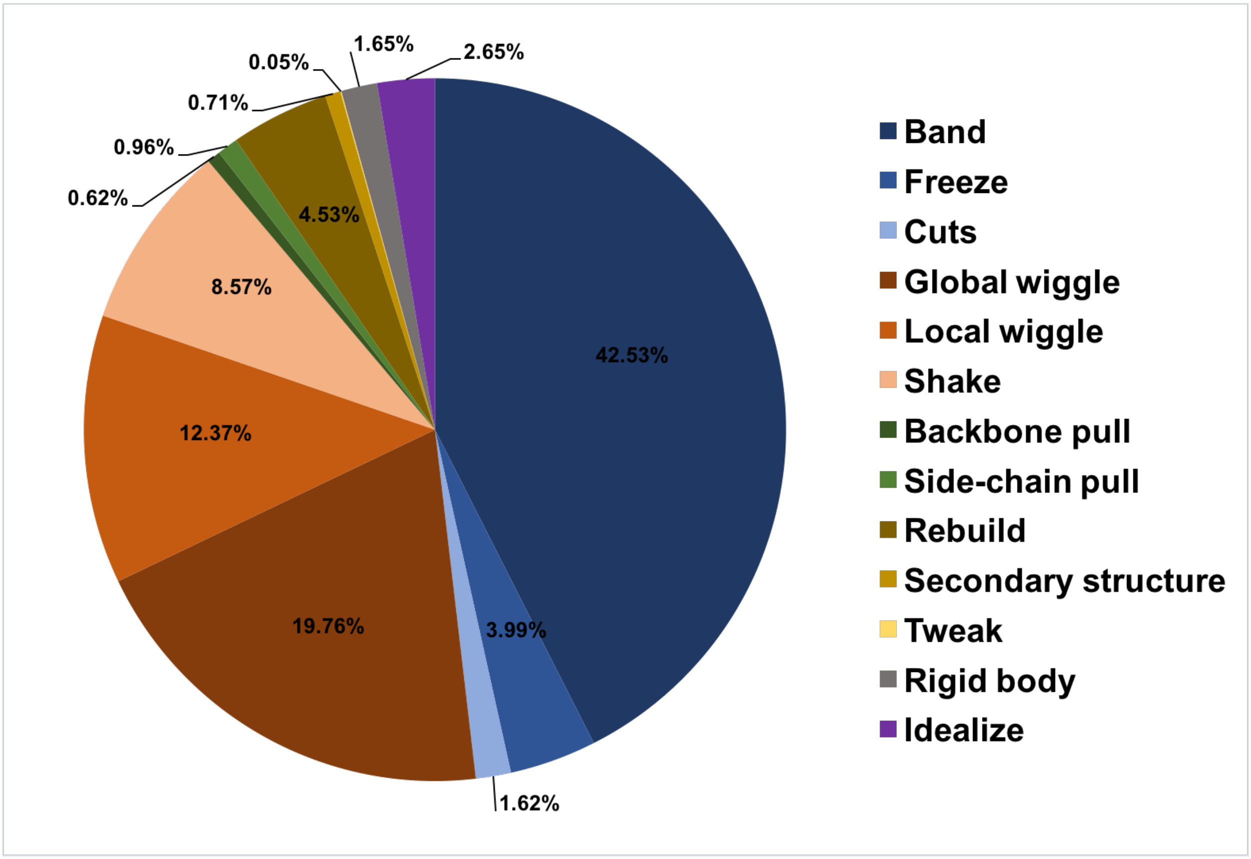

Supplementary Figure 3 | Total counts of move types used by Foldit players while creating the winning structure. Band, freeze, and cut actions (represented by shades of blue) adjust constraints. Global wiggle, local wiggle, and shake (represented by shades of orange) are automated optimizations of the protein. Backbone pull and side-chain pull actions (represented by shades of green) are move types in which players make manual adjustments to the protein by pulling directly on the structure. Rebuild, secondary structure, and tweak move types (represented by shades of yellow) are tools to modify or operate on secondary structures. Idealize actions (purple) set lengths and angles to their ideal values, and rigid body actions (gray) translate and rotate the protein. For additional explanations of the moves, see player-produced Foldit documentation online (http://foldit.wikia.com/wiki/Tools) and previous descriptions[1].

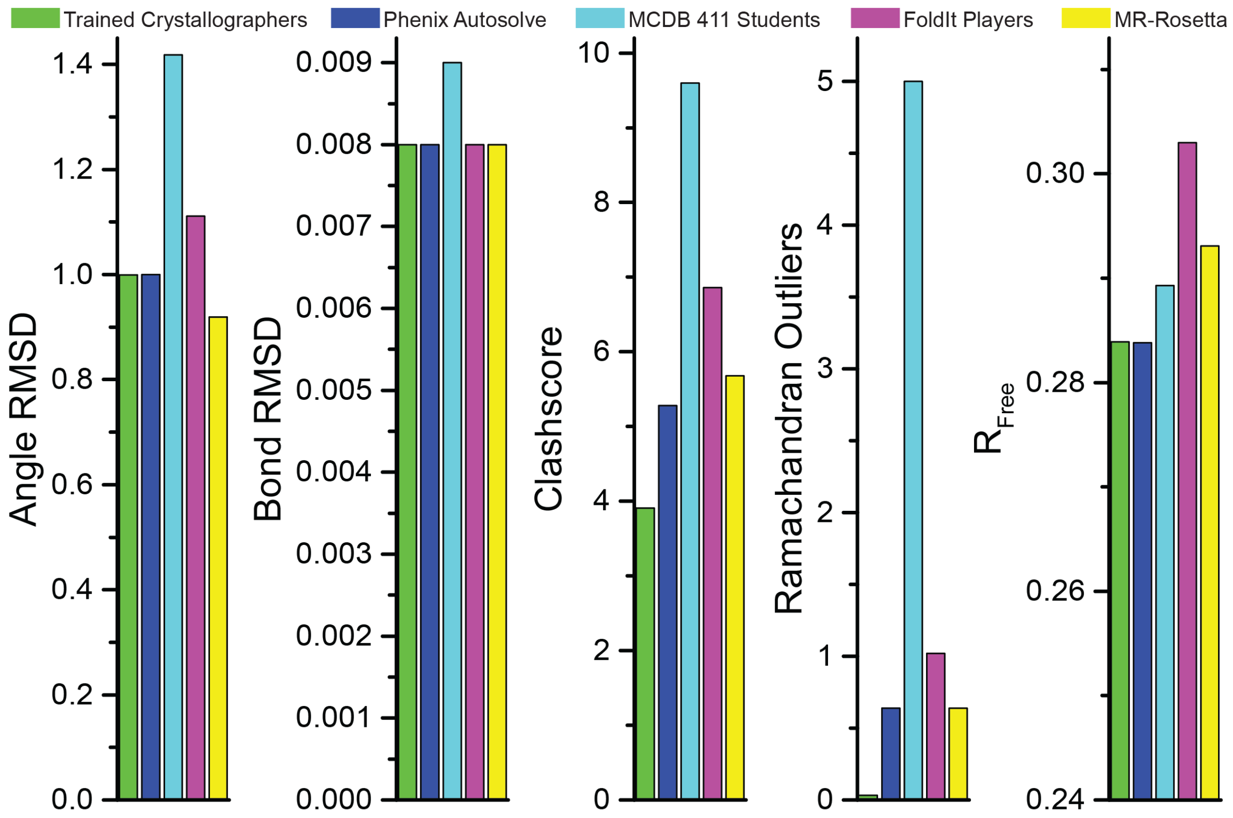

Supplementary Figure 4 | Comparison of key statistics of best models from each group before pruning disordered residues from Foldit structures. In all cases, lower values represent better scores. See Fig. 2 for key statistics after pruning disordered residues from the Foldit models.

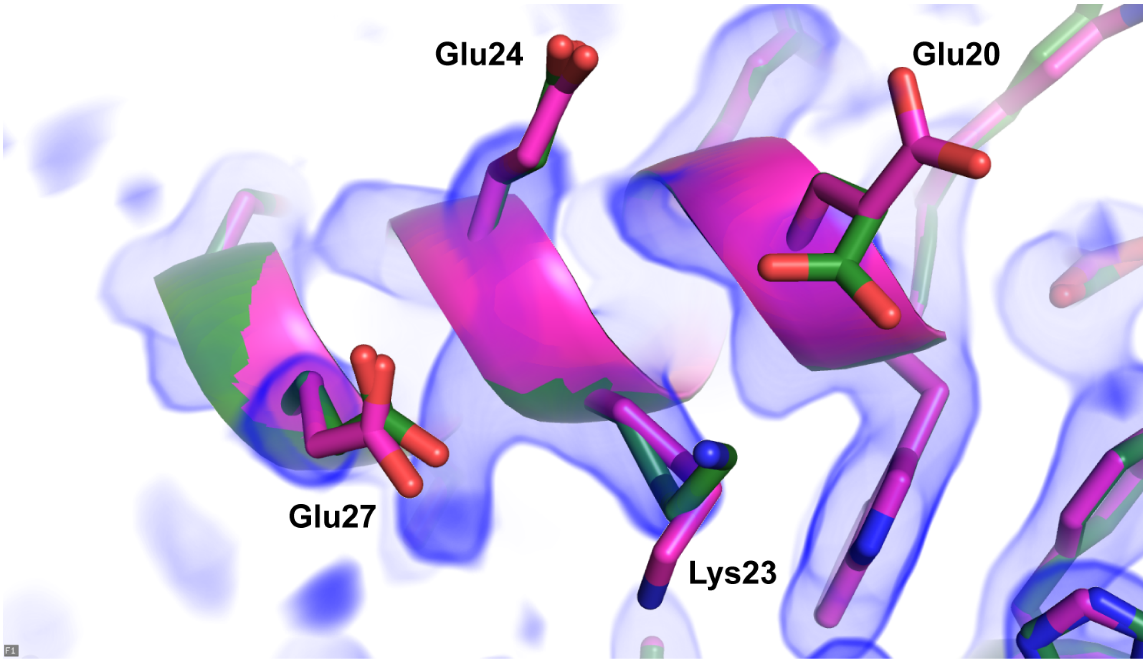

Supplementary Figure 5 | Example side chain conformational differences between the best Foldit model (green) and the best crystallographer-produced model (magenta). Map used in competition rendered as blue volume contoured at 0.8 σ.

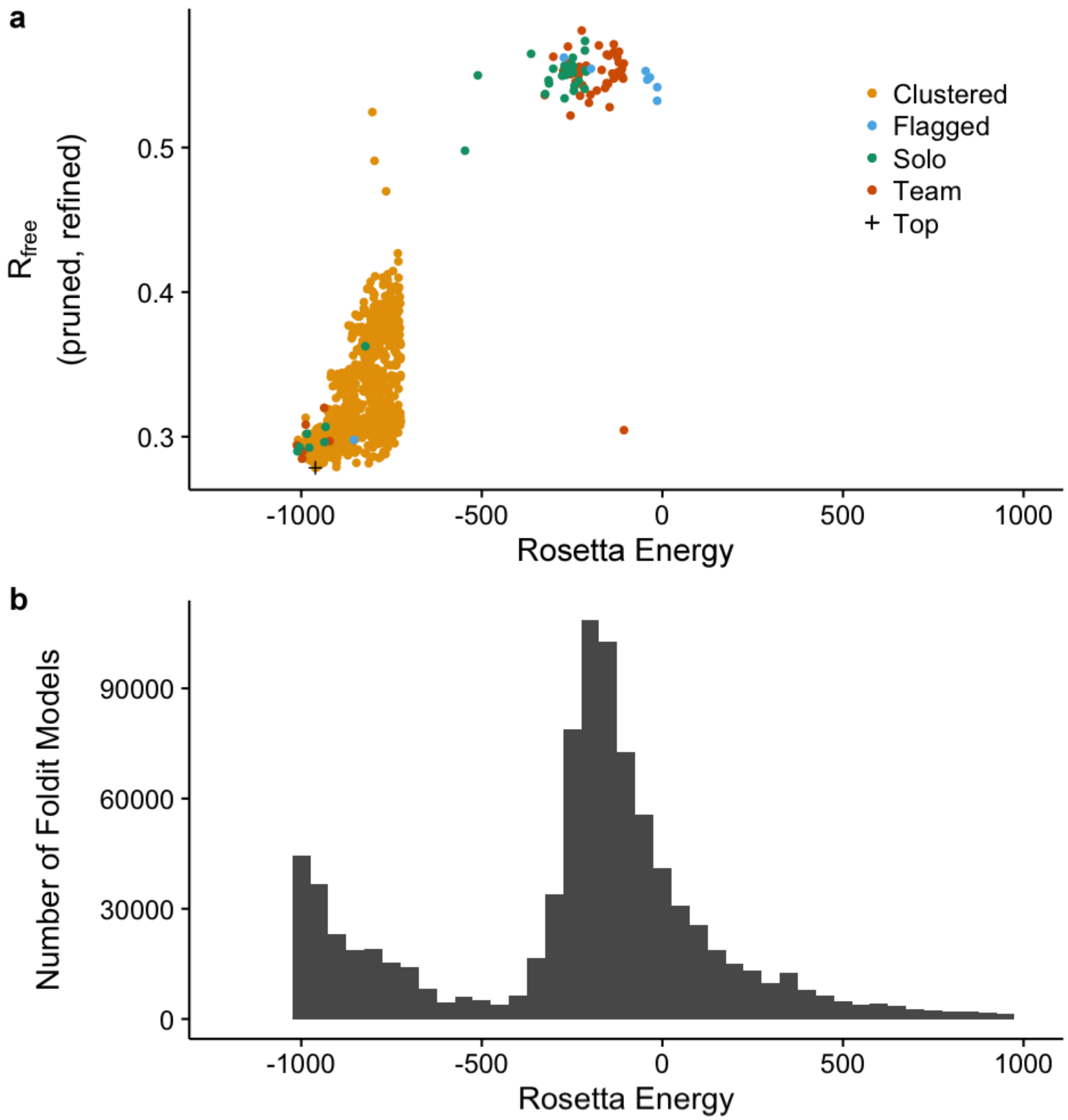

Supplementary Figure 6 | Foldit model score distribution and relation to *R*free. (a) After clustering the >900,000 Foldit models by structural alignment (with a cluster radius of 1 Å

RMSD), Phenix refinement was carried out with the 1000 best-scoring cluster centers, along with the 50 best-scoring solo or team models and any models that were flagged by Foldit players for special consideration—1094 Foldit models in total. The most favorable Foldit scores (lowest Rosetta energy) were associated with low R_{free} values. (**b**) The score distribution of Foldit models is strongly bimodal, suggesting that Rosetta scoring can effectively discriminate models that correctly fit the electron density map. Experienced Foldit players performed best in this puzzle. Among all 469 participants of the Foldit puzzle, each player had previously played an average of 10 Foldit puzzles with electron density; among the seven soloist players that achieved a Rosetta energy less than -700, each player averaged 39 previous electron density puzzles.

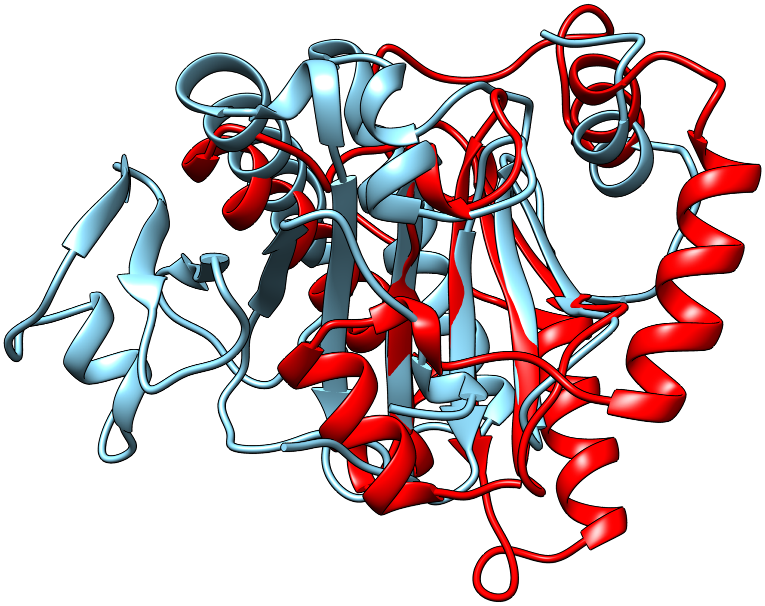

Supplementary Figure 7 | Structural alignment of HTC1 (red) with top DALI match, PDB 4EGU (blue).

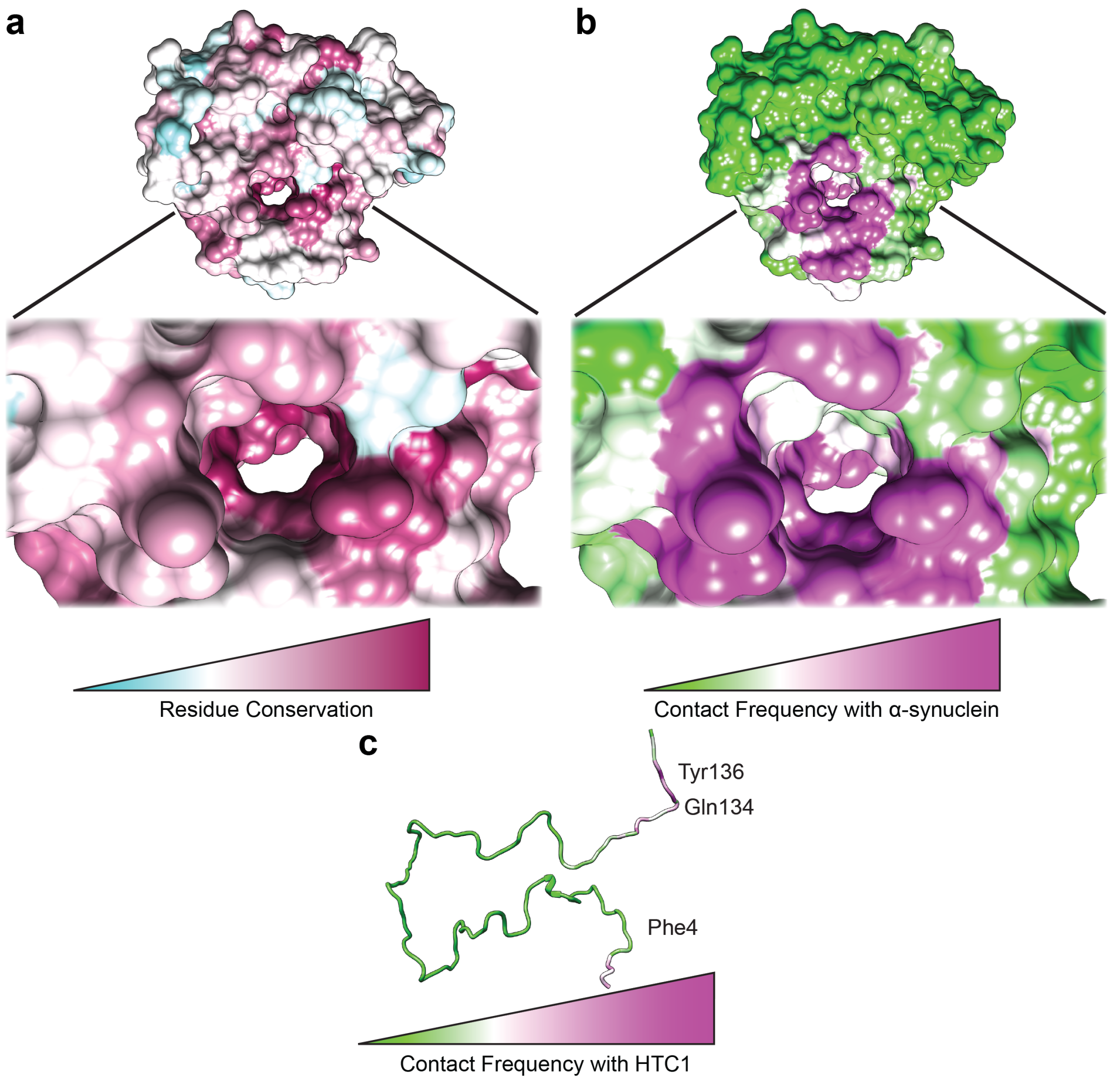

Supplementary Figure 8 | Docking simulations suggest that the highly conserved channel in HTC binds α-synuclein. Residue conservation (**a**) and projected contact map of docking simulations between HTC1 and α-synuclein onto surface of HTC1 (**b**). (**c**) Projected contact map of docking simulations between HTC1 and α-synuclein onto surface of α-synuclein. Contact frequency is ranked from dark green (lowest) to dark purple (highest), and residue conservation is ranked from cyan (lowest) to dark purple (highest).

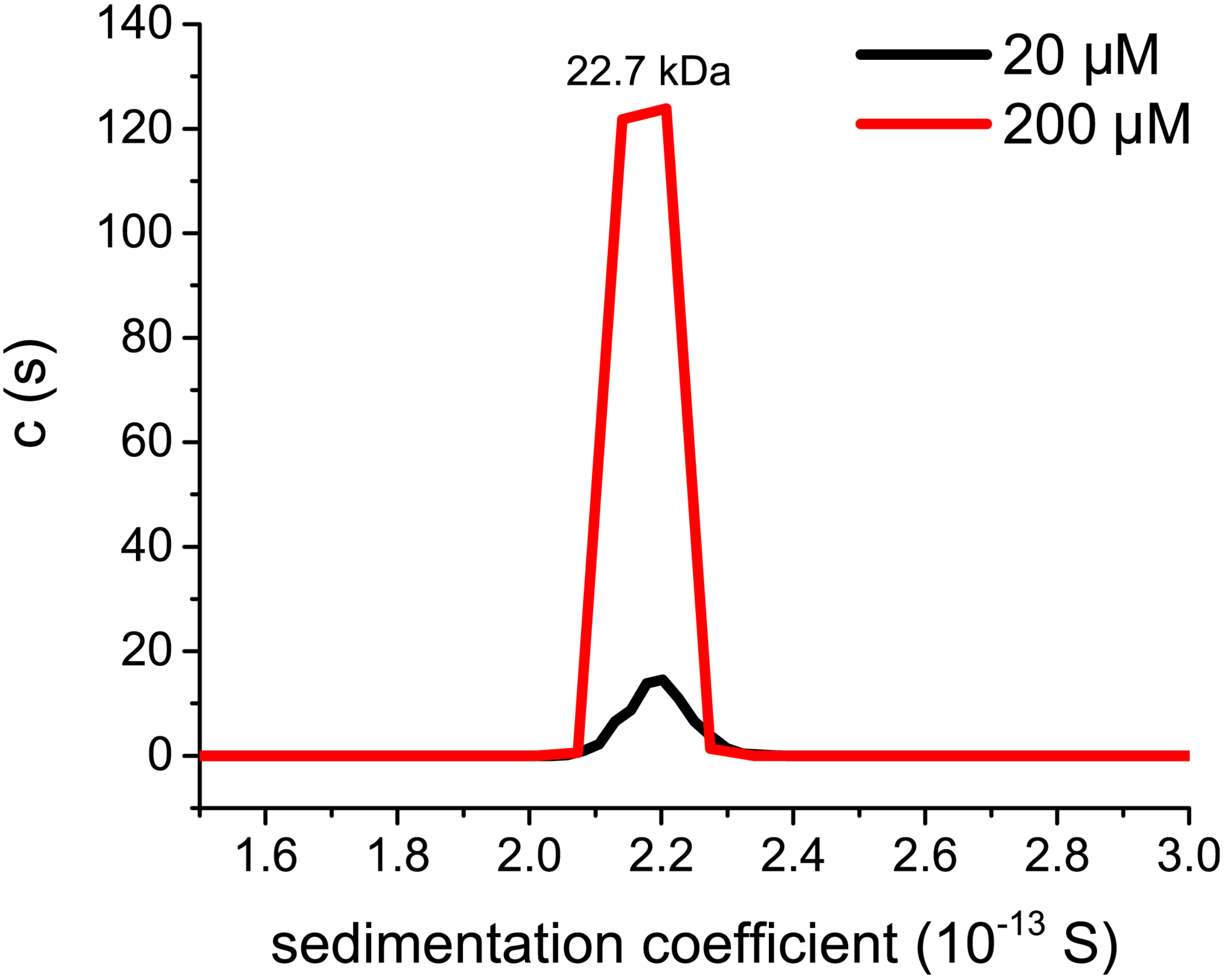

Supplementary Figure 9 | Sedimentation velocity analytical ultracentrifugation of 20 (black) and 200 (red) μM HTC1. Calculated molecular weight based on sequence: 22.9 kDa.

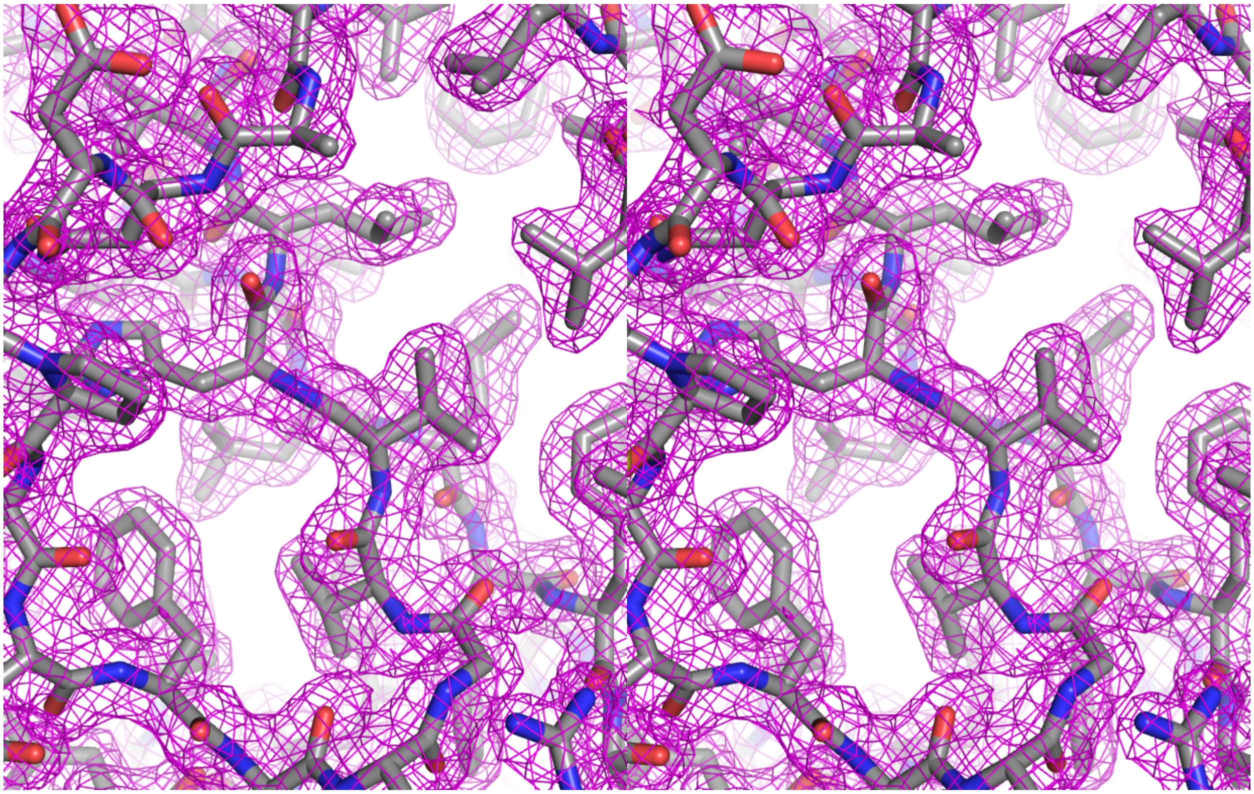

Supplementary Figure 10 | HTC1 refined density. $2mF_o$-DF_c density contoured at 1.0 σ (magenta) surrounding a portion of the final HTC1 structure, rendered in stereo.

Supplementary Table 1 | Top matches from DALI search of YPL067C

Chain	Z-score	RMSD	% Sequence identity	PDB description
4egu-A	4.9	3.6	11	HISTIDINE TRIAD (HIT) PROTEIN
4egu-B	4.8	3.6	10	HISTIDINE TRIAD (HIT) PROTEIN
5bv3-C	4.7	3.6	10	M7GPPPX DIPHOSPHATASE
4q61-B	4.6	3.2	16	UNCHARACTERIZED HIT-LIKE PROTEIN HP_0404
4zgl-F	4.6	3.3	16	UNCHARACTERIZED HIT-LIKE PROTEIN HP_0404
4zgl-D	4.6	3.3	16	UNCHARACTERIZED HIT-LIKE PROTEIN HP_0404
4q61-D	4.6	3.3	16	UNCHARACTERIZED HIT-LIKE PROTEIN HP_0404
4zgl-I	4.6	3.3	16	UNCHARACTERIZED HIT-LIKE PROTEIN HP_0404
4q61-I	4.6	3.3	16	UNCHARACTERIZED HIT-LIKE PROTEIN HP_0404
1xqu-B	4.5	3.7	12	HIT FAMILY HYDROLASE
1xqu-A	4.5	3.8	12	HIT FAMILY HYDROLASE
4q61-F	4.5	3.3	16	UNCHARACTERIZED HIT-LIKE PROTEIN HP_0404
4zgl-B	4.5	3.3	16	UNCHARACTERIZED HIT-LIKE PROTEIN HP_0404
4zgl-H	4.5	3.3	16	UNCHARACTERIZED HIT-LIKE PROTEIN HP_0404
4zgl-G	4.5	3.3	16	UNCHARACTERIZED HIT-LIKE PROTEIN HP_0404
4zgl-C	4.5	3.2	16	UNCHARACTERIZED HIT-LIKE PROTEIN HP_0404
4zgl-A	4.5	3.2	16	UNCHARACTERIZED HIT-LIKE PROTEIN HP_0404
4zgl-J	4.5	3.3	16	UNCHARACTERIZED HIT-LIKE PROTEIN HP_0404
4q61-C	4.5	3.2	16	UNCHARACTERIZED HIT-LIKE PROTEIN HP_0404
4q61-A	4.5	3.3	16	UNCHARACTERIZED HIT-LIKE PROTEIN HP_0404
5bv3-A	4.5	3.6	12	M7GPPPX DIPHOSPHATASE
4njz-C	4.4	4	17	HISTIDINE TRIAD NUCLEOTIDE-BINDING PROTEIN 2
3omf-A	4.4	3.5	14	PUTATIVE HISTIDINE TRIAD FAMILY PROTEIN
3oj7-A	4.4	3.5	15	PUTATIVE HISTIDINE TRIAD FAMILY PROTEIN
4q61-G	4.4	3.3	16	UNCHARACTERIZED HIT-LIKE PROTEIN HP_0404
4q61-J	4.4	3.3	16	UNCHARACTERIZED HIT-LIKE PROTEIN HP_0404
4q61-H	4.4	3.3	16	UNCHARACTERIZED HIT-LIKE PROTEIN HP_0404
4njx-A	4.3	4.1	18	HISTIDINE TRIAD NUCLEOTIDE-BINDING PROTEIN 2
3oxk-A	4.3	3.7	13	PUTATIVE HISTIDINE TRIAD FAMILY PROTEIN
5bv3-B	4.3	3.6	12	M7GPPPX DIPHOSPHATASE
4njx-B	4.3	4	18	HISTIDINE TRIAD NUCLEOTIDE-BINDING PROTEIN 2
3n1s-E	4.3	4.3	17	HIT-LIKE PROTEIN HINT
3n1s-M	4.3	4.1	17	HIT-LIKE PROTEIN HINT
4ini-B	4.3	3.9	18	HISTIDINE TRIAD NUCLEOTIDE-BINDING PROTEIN 2
4njz-B	4.3	4.1	17	HISTIDINE TRIAD NUCLEOTIDE-BINDING PROTEIN 2
4inc-B	4.3	3.9	18	HISTIDINE TRIAD NUCLEOTIDE-BINDING PROTEIN 2
4njz-A	4.3	3.9	18	HISTIDINE TRIAD NUCLEOTIDE-BINDING PROTEIN 2
4nk0-A	4.3	3.9	18	HISTIDINE TRIAD NUCLEOTIDE-BINDING PROTEIN 2
4njz-D	4.3	3.9	18	HISTIDINE TRIAD NUCLEOTIDE-BINDING PROTEIN 2
3r6f-A	4.2	3.6	14	HIT FAMILY PROTEIN
1xml-A	4.2	3.8	14	HEAT SHOCK-LIKE PROTEIN 1
3n1s-B	4.2	4.1	17	HIT-LIKE PROTEIN HINT
3n1s-F	4.2	4.1	17	HIT-LIKE PROTEIN HINT
3n1t-B	4.2	4	16	HIT-LIKE PROTEIN HINT
3n1t-E	4.2	4	16	HIT-LIKE PROTEIN HINT
3tw2-B	4.2	4	15	HISTIDINE TRIAD NUCLEOTIDE-BINDING PROTEIN 1
3n1t-A	4.2	3.9	16	HIT-LIKE PROTEIN HINT

Supplementary Note 1

We gave the Foldit players the same lectin scytovirin puzzle as the students from the previous iteration of the assignment to see if Foldit players could come up with similar solutions. Foldit players were provided with the same electron density map that the students had previously received and a polypeptide of five alanine residues in extended conformation for players to start with for model building. Players were allowed to add and mutate residues as needed, up to a length of 95 amino acids (the length of the native protein). No sequence information was given, and—unlike the class assignment—the position of the N-terminal amino acid was not provided. Although two weeks were allotted for the Foldit puzzle, players had reproduced the complete backbone of the native protein within 20 h of the puzzle's first posting. At many positions in top-ranked Foldit models, players preferred incorporating amino acids not not corresponding to the native sequence. Although they often diminished the fit-to-density, these substitutions were reinforced by other score terms in the Rosetta score function. For example, it was common for Foldit players to substitute a histidine at a solvent-exposed position actually occupied by Phe37, offsetting the drop in fit-to-density with a solvation bonus. At least two players produced accurate models with 100% sequence identity and subsequently flagged them for special consideration, presumably having identified the native protein by sequence or structural alignment. Similar to the undergraduate structures, both of these Foldit models ranked in the 100th percentile in both Molprobity clashscore and total score.

Of note, in the lectin scytovirin puzzle, the students and Foldit players were given density covering the whole unit cell, including density from multiple monomer proteins. The students nearly uniformly built into a single monomer chain (as opposed to bridging adjacent monomers), as did the top nine Foldit structures (Supplementary Fig. 2). As such, for planning future

assignments, we considered it unnecessary to provide the players and students with electron density outside of a single monomer. Given the relatively large size of the YPL067C Foldit puzzle compared with previous electron density puzzles, we trimmed the map around a single monomer to enhance software performance speed and facilitate player participation.

Supplementary Note 2

Lack of sequence similarity to YPL067C in PDB

When students were given an electron density map from a protein deposited in the PDB, a small proportion of the students used BLAST to find the structure. These students were then able to download the complete structure and use it as a guide in their model-building efforts. We had observed similar results with Foldit electron density puzzles that used published structures. To eliminate the potential for this type of cheating in the competition, we avoided using a published electron density map or any structure that was similar enough to any structure in the PDB to be discovered by using BLAST. The Critical Assessment of Structure Prediction (CASP) protein competition, where modellers compete to predict structures, also focuses on unpublished crystal structures for similar reasons[2]. The lowest E-value score of YPL067C to any protein in the PDB, as of February 15th, 2016, was 1.9. As only E-values < 0.005 are generally considered significant, competition participants were unable to find a YPL067C homologue in the PDB to direct modeling efforts. Similarly, a wide-search molecular replacement attempt[3], which uses structures of every known domain as molecular search models, did not successfully find a structure solution for the experimental electron density. The lack of structural information on YPL067C prevented cheating by the various model-building competitors.

Comparison before pruning disordered regions

Based on the comparison criteria before pruning disordered regions from the Foldit structures, the best structure came from the trained crystallographer group. When evaluating the best structure from each group, the trained crystallographers produced the structure that scored either the best or was tied for the best in nearly every category. Most notably, the best trained-crystallographer structure contained zero Ramachandran outliers and the lowest Molprobity clashscore (3.9). Interestingly, Phenix Autosolve produced the second highest quality structure, with the second lowest number of steric clashes, and essentially equivalent R_{free} values and RMSDs for angles and bonds as the trained crystallographer structures.

HTC1 structural details

The N-terminal 15 residues of the HTC1 protein are disordered and could not be modeled in either the SeMet or native datasets. In the native dataset, residues Met35 to Arg50 appear to form a loop and right-handed α-helix that are marked by high B-factors, which are not visible in the SeMet dataset used in the model-building competition. A second disordered region discernable in the native dataset but not the SeMet dataset includes the loop between residues Trp66 and Ala78.

Despite its similarity to HIT protein structure, there are differences between the structure of HTC1 and canonical HIT proteins. The majority of the top DALI search results (Supplementary Table 1) dimerize through an antiparallel ß-sheet interaction (Supplementary Fig. 7). In contrast, HTC1 is monomeric in the asymmetric unit (Fig. 3) and in solution (Supplementary Fig. 9). Additionally, the sequence organization of a standard histidine triad is His-x-His-x-His, where x are hydrophobic residues. Instead of being distributed over a stretch of 5 amino acids, the histidine triad members in this newly recognized HTC family are distributed over a stretch 60 amino acids. It therefore appears that HTC1 is a somewhat atypical member of the HIT protein

superfamily. Indeed, the protein family servers Pfam[4] and Interpro[5] currently list YPL067C as a member of a distinct family of unknown function (DUF3605), with over 900 members found in a wide variety of eukaryotes and viruses.

We examined the structure of HTC1 for clues as to the mechanism of its anti-aggregation activity. There are various conserved surface features of HTC1 that could possibly be involved in binding peptides, including a shallow channel running near the surface of the protein (Supplementary Fig. 8a). This shallow channel is ~4.3 Å in width at one end, narrowing as it passes through the protein. This channel is therefore large enough to potentially accommodate an unfolded protein terminus binding to HTC1. Blind docking of the α-synuclein NMR ensemble[6] with HTC1 suggests that this highly conserved channel is the most likely α-synuclein binding site (Supplementary Fig. 8b). Thus, it is possible that HTC1 prevents amyloid aggregation by binding protein termini in its conserved channel. This channel appears to be unique to the HTC family, as none of the top ten unique DALI hits in the PDB (Supplementary Table 1) feature a corresponding channel.

In the final model, a large tetrahedral molecule sits at the center of the channel, near His166 and His168 and the side chain of Gln154. Given the high concentration of sulfate ions in the crystallization buffer, it is highly likely this density arises from a bound sulfate ion. Several of the top DALI search hits also were crystallized with bound sulfate ions. However, at this resolution, we cannot entirely rule out that this density could be due to a bound phosphate carried over from purification buffers. This ion occupies the only position in the channel that is well-segregated from bulk solution. The closest DALI search hit, 4EGU, was crystallized bound to guanosine monophosphate. Analogous electron density for a potential nucleotide bound in the correlating site of HTC1 is poorly defined, perhaps due to partial occupancy, and also could

represent a glycerol molecule from the crystallization solution.

Supplementary References

1. Cooper S*, et al.* Predicting protein structures with a multiplayer online game. *Nature* **466**, 756-760 (2010).

2. Moult J. The current state of the art in protein structure prediction. *Curr Opin Biotech* **7**, 422-427 (1996).

3. Stokes-Rees I, Sliz P. Protein structure determination by exhaustive search of Protein Data Bank derived databases. *Proc Natl Acad Sci U S A* **107**, 21476-21481 (2010).

4. Finn RD*, et al.* The Pfam protein families database: towards a more sustainable future. *Nucleic Acids Res* **44**, D279-285 (2016).

5. Mitchell A*, et al.* The InterPro protein families database: the classification resource after 15 years. *Nucleic Acids Res* **43**, D213-221 (2015).

6. Schwalbe M*, et al.* Predictive atomic resolution descriptions of intrinsically disordered hTau40 and alpha-synuclein in solution from NMR and small angle scattering. *Structure* **22**, 238-249 (2014).
